# Post-CCRT pelvic lymph node size and pathological nodal involvement in cervical cancer

**DOI:** 10.1007/s10147-026-03174-0

**Published:** 2026-08-19

**Authors:** Teppei Suzuki, Nobuhisa Yoshikawa, Chitose Kamiya, Komei Katayama, Kazumasa Mogi, Shohei Iyoshi, Kosuke Yoshida, Masato Yoshihara, Yukari Nagao, Satoshi Tamauchi, Akira Yokoi, Kaoru Niimi, Hiroaki Kajiyama

**Affiliations:** 1 Department of Obstetrics and Gynecology, Okazaki City Hospital, Okazaki, Japan; 2 Department of Obstetrics and Gynecology, Nagoya University Graduate School of Medicine, 65 Tsurumai-cho, Showa- ku, Nagoya, 466-8550 Japan

**Keywords:** Cervical cancer, Concurrent chemoradiotherapy, Lymph node metastasis, Computed tomography, ROC analysis, Pathological response

## Abstract

**Background:**

Lymph node metastasis is an important prognostic factor in cervical cancer, but assessment of nodal response after concurrent chemoradiotherapy (CCRT) remains difficult. This study evaluated the diagnostic value of post-CCRT lymph node size for predicting pathological nodal involvement using a cohort treated with CCRT followed by planned surgery.

**Methods:**

This retrospective cohort study included 57 patients with cervical cancer who underwent CCRT followed by surgery including pelvic lymphadenectomy. The short- and long-axis diameters of the largest pelvic lymph node were measured on computed tomography before and after CCRT. Histopathological findings of resected lymph nodes were used as the reference standard. Receiver operating characteristic (ROC) analysis was performed to assess diagnostic performance.

**Results:**

Pathological nodal metastasis was identified in 17 of 57 patients (29.8%). Post-CCRT lymph node diameters were significantly larger in the ypN1 group than in the ypN0 group. The short-axis diameter showed better diagnostic performance than the long-axis diameter, with an area under the curve of 0.859 (95% CI, 0.752–0.966) versus 0.802 (95% CI, 0.676–0.927). Among integer cutoff values, the short-axis diameter showed the highest Youden index at 5 mm and the highest F1 score at 7 mm. At a 7-mm cutoff, sensitivity, specificity, positive predictive value, negative predictive value, and accuracy were 58.8%, 90.0%, 71.4%, 83.7%, and 80.7%, respectively.

**Conclusion:**

Post-CCRT pelvic lymph node size, especially the short-axis diameter, may be useful for estimating residual pathological nodal disease in cervical cancer.

## Introduction

Cervical cancer remains an important gynecologic malignancy worldwide, particularly in regions with limited access to human papillomavirus vaccination, screening, and timely treatment [1]. According to recent global estimates, cervical cancer remains the fourth most common cancer in women worldwide and disproportionately affects low- and middle-income countries. For patients with locally advanced cervical cancer, concurrent chemoradiotherapy (CCRT) remains the standard treatment, and current international guidelines do not recommend routine completion surgery after chemoradiation because its survival benefit has not been clearly established and treatment-related morbidity remains a concern [2, 3].

Among the prognostic factors in cervical cancer, lymph node metastasis has particular clinical importance because it is closely associated with recurrence and survival [4, 5]. The importance of nodal involvement was reflected in the 2018 International Federation of Gynecology and Obstetrics (FIGO) staging revision, which formally integrated nodal involvement into stage IIIC, reflecting the need to evaluate nodal disease more precisely in routine practice [6]. Assessment of nodal status is important not only at initial staging but also after treatment, because residual nodal disease may affect follow-up planning, additional diagnostic evaluation, and decisions regarding local salvage strategies [7].

In daily practice, however, post-treatment evaluation of lymph nodes is often difficult. Residual nodal enlargement after CCRT does not necessarily indicate viable tumor, because radiation-induced fibrosis, edema, necrosis, and inflammatory change may persist on imaging [8]. Conversely, metastatic foci may remain in lymph nodes that have become small after treatment [9]. This mismatch between radiologic response and pathological status makes post-CCRT nodal assessment difficult [10]. Recent imaging studies and reviews have shown that although magnetic resonance imaging (MRI) and computed tomography (CT) are useful for response assessment, neither modality is perfect in distinguishing treatment-related change from residual disease, particularly in the early post-treatment setting [11, 12].

This problem is clinically important because residual disease after chemoradiation may lead to consideration of biopsy or salvage surgery, yet radiologic suspicion alone is not always reliable. A systematic review of salvage surgery after chemoradiation reported that when surgery was performed based only on radiologic suspicion, pathological residual disease was confirmed in only a subset of cases, highlighting the risk of unnecessary surgery and procedure-related complications [13]. Persistent lymph node abnormalities after definitive chemoradiotherapy have also been associated with poorer survival, underscoring the importance of identifying patients at higher risk of residual nodal disease [14].

At our institution, some patients with cervical cancer were historically treated with CCRT followed by planned surgery including pelvic lymphadenectomy. Although this strategy is different from current standard management, it allowed direct comparison between post-CCRT imaging findings and pathological nodal status. While the previous institutional report mainly evaluated the role of additional hysterectomy after chemoradiation in terms of survival and recurrence outcomes [15], the present study was undertaken to determine whether post-treatment pelvic lymph node size on CT could predict residual pathological nodal metastasis. Previous clinical studies have suggested that nodal size itself has prognostic and therapeutic relevance in cervical cancer, but data specifically addressing the diagnostic value of post-CCRT lymph node size with pathological confirmation remain limited [16, 17].

Therefore, we investigated whether pelvic lymph node size on computed tomography (CT) after CCRT could predict pathological nodal involvement in patients with cervical cancer who subsequently underwent surgery with lymphadenectomy. We focused on both short-axis and long-axis diameters of the largest pelvic lymph node and evaluated their diagnostic performance for identifying residual nodal metastasis. We also explored clinically useful cutoff values for interpreting post-treatment nodal findings in patients with suspected persistent disease after CCRT.

## Patients and methods

### Study design and patient population

This was a single-institution retrospective cohort study. After approval by the institutional review board, the medical records of 1,080 patients diagnosed with cervical cancer at our institution between April 2005 and August 2021 were reviewed. Among them, 77 patients who underwent CCRT followed by planned surgery were identified. After exclusion of 20 patients because of incomplete clinical or imaging data, 57 patients were included in the final analysis. Eligible patients had histologically confirmed cervical cancer, completed CCRT, and subsequently underwent surgery including hysterectomy and pelvic lymphadenectomy. Because this cohort was derived from a historical series treated over a long study period, disease stage was classified according to the FIGO 2008 staging system. The final study population included patients with stage IB1 to IIIB disease. Part of this historical cohort overlapped with a previously reported institutional series evaluating the clinical role of additional hysterectomy after chemoradiation [15].

### Treatment protocol

Patients in this cohort were treated according to the institutional practice used during the study period, in which surgery was planned after CCRT. The aim of this protocol was to enhance local disease control by combining chemoradiotherapy with surgery and, at the same time, to decrease the invasiveness and potential morbidity of surgery by reducing tumor volume before operation. External beam radiation therapy (EBRT) was principally delivered to the whole pelvis at a total dose of 39.6–50.4 Gy in 22–28 fractions. In patients with insufficient reduction of the primary tumor after EBRT, brachytherapy was additionally performed at the discretion of the treating physicians. In principle, concurrent chemotherapy was composed of 2 courses of cisplatin (70 mg/m^2^ on day 1) and 5‑fluorouracil (700 mg/m^2^, 24 h continuous intravenous infusion on days 1–4) combination chemotherapy. After completion of radiotherapy, surgery including hysterectomy and pelvic lymphadenectomy was performed. Because this treatment strategy was used in a historical cohort, it differs from current standard management for locally advanced cervical cancer.

### Imaging protocol and nodal measurement

In principle, patients underwent contrast-enhanced CT before and after CCRT. When CT images were unavailable, MRI was used as an alternative for nodal measurement. For post-treatment nodal assessment, CT was used in 43 patients and MRI was used in 14 patients. Both the short-axis and long-axis diameters of the largest pelvic lymph node were measured on pre- and post-treatment images by gynecologic oncologists who were blinded to the pathological results. Post-treatment imaging was performed during the preoperative assessment period. The median interval from completion of CCRT to surgery was 26 days (interquartile range [IQR], 20–33 days; range, 8–82 days), and the median interval from post-treatment imaging to surgery was 16 days (IQR, 8–25 days; range, 2–60 days). Pre-treatment clinical nodal status (cN0 or cN1) was determined by the treating physicians at the time of initial diagnosis based on the available pre-treatment imaging findings, including contrast-enhanced CT and/or MRI, and was extracted from the documentation in the medical records.

### Histopathological evaluation

All patients underwent pelvic lymphadenectomy at the time of surgery. Resected lymph nodes were examined histopathologically, and the presence of residual viable tumor cells was defined as pathological nodal metastasis. Histopathological nodal status was used as the reference standard for this study.

### Statistical analysis

Clinicopathological variables were compared between the pathological nodal metastasis-negative and -positive groups. Continuous variables in the between-group analysis were compared using the Mann–Whitney U test. Categorical variables were analyzed using the chi-squared test or Fisher’s exact test, as appropriate. Statistical significance was set at *p* < 0.05. Receiver operating characteristic (ROC) curve analysis was performed to evaluate the diagnostic performance of post-CCRT lymph node size for predicting pathological nodal metastasis. The area under the curve (AUC) was calculated for both short-axis and long-axis diameters. Sensitivity, specificity, positive predictive value, negative predictive value, and accuracy were calculated for selected cutoff values. All statistical analyses were performed using GraphPad Prism version 10 (GraphPad Software, Boston, MA, USA).

## Results

The final study cohort comprised 57 patients, with a median age of 52 years (range, 28–70 years). The most common stage was FIGO 2008 stage IIB (36/57, 63.2%), followed by stages IB2 and IIA2 (8/57 each, 14.0%). Histologically, squamous cell carcinoma was the most frequent subtype (27/57, 47.4%), followed by adenocarcinoma (24/57, 42.1%). After CCRT and subsequent surgery, 17 patients (29.8%) were diagnosed with pathological lymph node metastasis (ypN1), whereas 40 patients (70.2%) were classified as ypN0. As shown in Table 1, there were no significant differences between the ypN0 and ypN1 groups in age, stage, histological subtype, radiotherapy regimen, chemotherapy regimen, or treatment timing variables. In contrast, pre-treatment clinical nodal status differed significantly between the two groups, with cN1 disease being more frequent in the ypN1 group than in the ypN0 group (*p* = 0.034). Recurrence was more frequent in the ypN1 group than in the ypN0 group, although the difference did not reach statistical significance (41.2% vs. 20.0%, *p* = 0.113).

**Table 1:** Patient and Tumor Characteristics Stratified by Pathological Nodal Status

Characteristic	Overall (*n* = 57)	ypN0 (*n* = 40)	ypN1 (*n* = 17)	*p* value
Age years (median, range)	52 (28–70)	50 (28–70)	52 (31–66)	0.827
CCRT completion to surgery, days (median, range)	26 (8–82)	25 (8–82)	26 (12–43)	0.979
Post-treatment imaging to surgery (median, range)	16 (2–60)	16 (2–60)	15 (4–41)	0.958
*Stage*				
IB1	1 (1.8%)	0 (0.0%)	1 (5.9%)	0.236
IB2	8 (14.0%)	4 (10.0%)	4 (23.5%)	
IIA1	2 (3.5%)	1 (2.5%)	1 (5.9%)	
IIA2	8 (14.0%)	6 (15.0%)	2 (11.8%)	
IIB	36 (63.2%)	27 (67.5%)	9 (52.9%)	
IIIB	2 (3.5%)	2 (5.0%)	0 (0.0%)	
*Histology*				
Squamous cell carcinoma	27 (47.4%)	21 (52.5%)	6 (35.3%)	0.200
Adenocarcinoma	24 (42.1%)	17 (42.5%)	7 (41.2%)	
Adenosquamous carcinoma	3 (5.3%)	1 (2.5%)	2 (11.8%)	
Others	3 (5.3%)	1 (2.5%)	2 (11.8%)	
*Radiotherapy*				
Whole-pelvic RT 39.6 Gy	43 (75.4%)	32 (80.0%)	11 (64.7%)	0.084
Whole-pelvic RT < 39.6 Gy	5 (8.8%)	1 (2.5%)	4 (23.5%)	
Whole-pelvic RT 50.4 Gy	5 (8.8%)	4 (10.0%)	1 (5.9%)	
Whole-pelvic RT + brachytherapy	4 (7.0%)	3 (7.5%)	1 (5.9%)	
*Chemotherapy*				
PF, 2 cycles	43 (75.4%)	31 (77.5%)	12 (70.6%)	0.073
PF, 3 cycles	4 (7.0%)	4 (10.0%)	0 (0.0%)	
PF, 4 cycles	3 (5.3%)	2 (5.0%)	1 (5.9%)	
PF, 5 cycles	2 (3.5%)	2 (5.0%)	0 (0.0%)	
EP, 2 cycles	2 (3.5%)	1 (2.5%)	1 (5.9%)	
Other regimens	3 (5.3%)	0 (0.0%)	3 (17.6%)	
*Clinical node status at diagnosis*				
cN0	23 (40.4%)	20 (50.0%)	3 (17.6%)	0.034
cN1	31 (54.4%)	18 (45.0%)	13 (76.5%)	
Unknown	3 (5.3%)	2 (5.0%)	1 (5.9%)	
*Recurrence*				
Yes	15 (26.3%)	8 (20.0%)	7 (41.2%)	0.113
No	42 (73.7%)	32 (80.0%)	10 (58.8%)	

*Abbreviations: RT*,* radiotherapy; PF*,* cisplatin + fluorouracil-based regimen; EP*,* etoposide + cisplatin.*

**Fig. 1:**
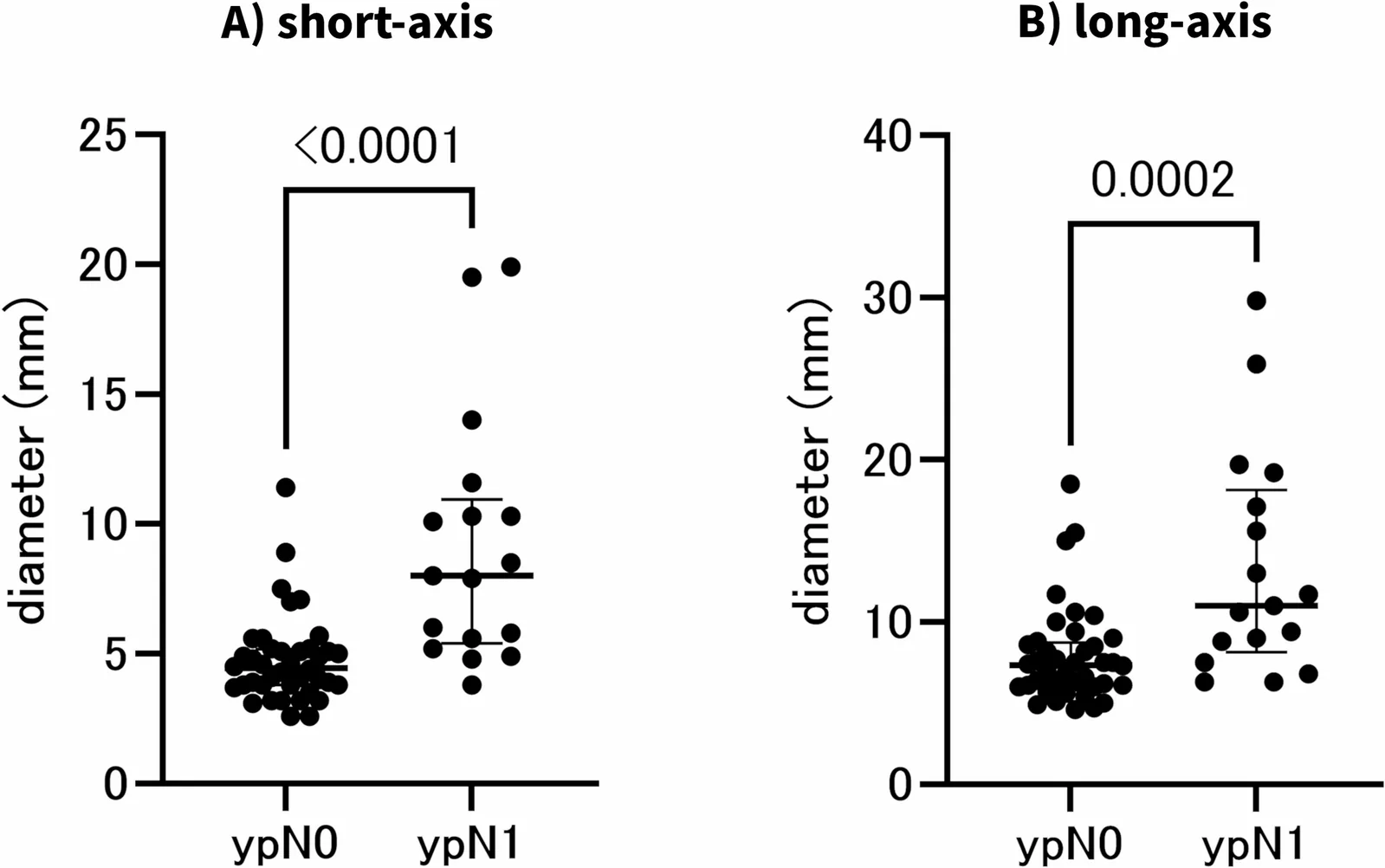
Comparison of post-CCRT pelvic lymph node diameters between patients with (ypN1, *n* = 17) and without (ypN0, *n* = 40) pathological nodal metastasis. **A** Short-axis diameter. **B** Long-axis diameter. Each dot represents the largest pelvic lymph node of an individual patient. Horizontal bars indicate the median and interquartile range. *p* values were calculated using the Mann–Whitney U test

We next compared post-CCRT lymph node size between the ypN0 and ypN1 groups. As shown in Fig. 1, the largest pelvic lymph node remaining after treatment was significantly larger in the ypN1 group than in the ypN0 group for both the short-axis and long-axis diameters. In addition, we re-evaluated all 17 patients with ypN1 disease to assess whether the largest lymph node identified on post-treatment imaging corresponded anatomically to the site of pathological nodal metastasis. In all 17 ypN1 patients, the largest lymph node on post-treatment imaging was located in the same anatomical region as the pathologically positive lymph node.

**Fig. 2:**
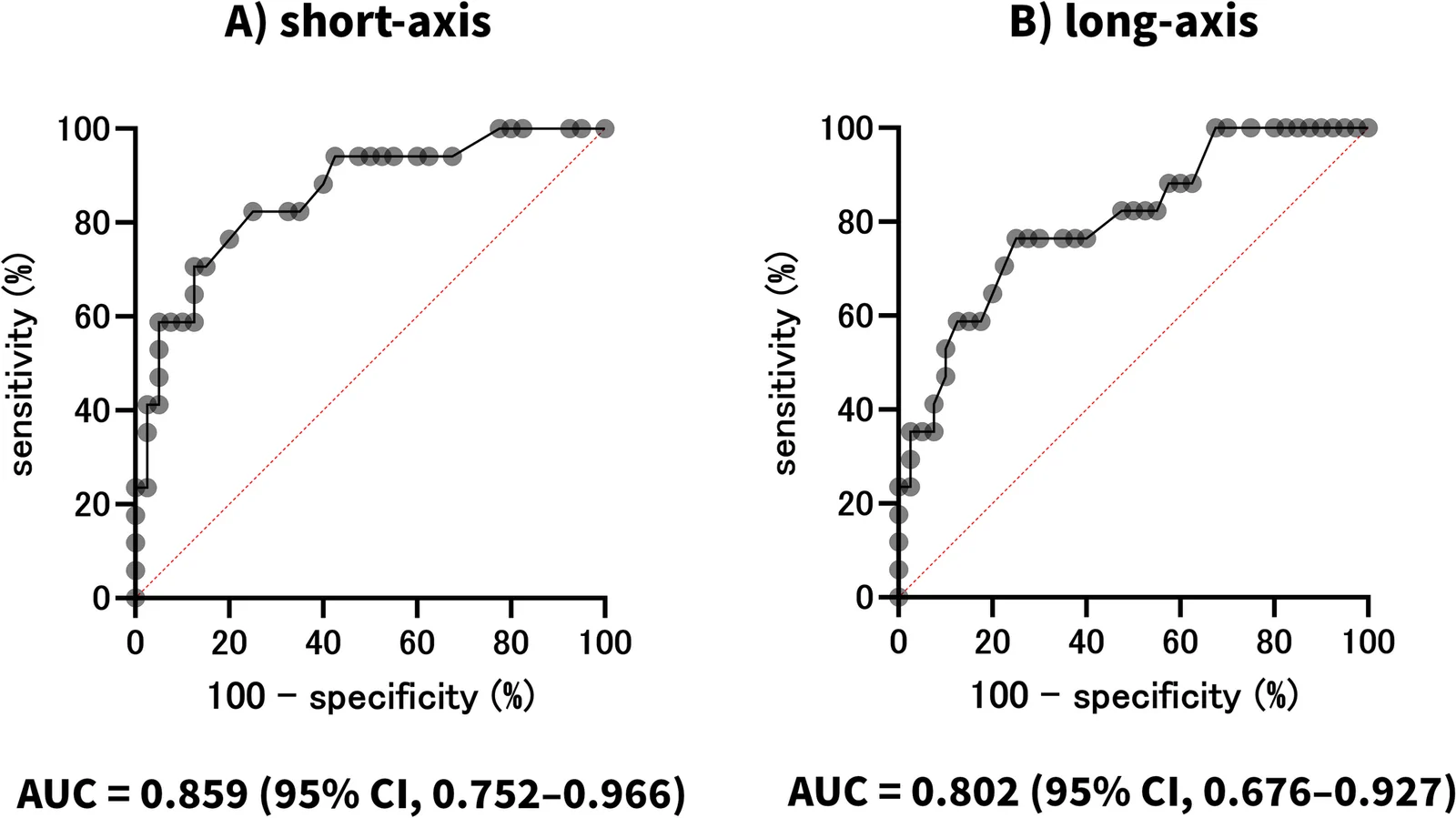
Receiver operating characteristic curves of post-CCRT pelvic lymph node diameters for predicting pathological nodal metastasis. **A** Short-axis diameter (AUC = 0.859; 95% CI, 0.752–0.966). **B** Long-axis diameter (AUC = 0.802; 95% CI, 0.676–0.927). The dashed diagonal line represents the reference line of no discrimination

ROC curve analysis was then performed to evaluate the diagnostic performance of post-treatment lymph node size for predicting pathological nodal involvement (Fig. 2). The AUC for the post-CCRT short-axis diameter was 0.859 (95% CI, 0.752–0.966), whereas that for the long-axis diameter was 0.802 (95% CI, 0.676–0.927). The diagnostic indices for representative integer cutoff values are summarized in Table 2. Among the examined short-axis thresholds, the highest Youden index was observed at 5 mm (0.499), whereas the highest F1 score was observed at 7 mm (0.645). Among the examined long-axis thresholds, the highest Youden index was observed at 9 mm (0.447).

**Table 2:** Diagnostic performance of post-CCRT pelvic lymph node diameters at integer cut-off values for predicting pathological nodal metastasis

Variable	Cut-off, mm	Sensitivity, %	Specificity, %	PPV, %	NPV, %	Accuracy, %	Youden index	F1 score
Short-axis diameter	4	94.1	40.0	40.0	94.1	56.1	0.341	0.561
	5	82.4	67.5	51.9	90.0	71.9	0.499	0.636
	6	58.8	87.5	66.7	83.3	78.9	0.463	0.625
	7	58.8	90.0	71.4	83.7	80.7	0.488	0.645
	8	47.1	95.0	80.0	80.9	80.7	0.421	0.593
Long-axis diameter	6	100.0	25.0	36.2	100.0	47.4	0.250	0.531
	7	82.4	47.5	40.0	86.4	57.9	0.299	0.538
	8	76.5	65.0	48.1	86.7	68.4	0.415	0.591
	9	64.7	80.0	57.9	84.2	75.4	0.447	0.611
	10	58.8	85.0	62.5	82.9	77.2	0.438	0.606

*PPV*,* positive predictive value; NPV*,* negative predictive value*

## Discussion

The present study examined whether residual nodal disease after CCRT could be estimated from post-treatment pelvic lymph node size. This study was based on a historical institutional strategy in which surgery, including pelvic lymphadenectomy, was planned after CCRT. This treatment sequence differs from the current standard approach for locally advanced cervical cancer, in which definitive chemoradiotherapy with image-guided adaptive brachytherapy (IGABT) is generally used without planned surgery. Therefore, the present findings should not be interpreted as directly applicable cutoff values for definitive CCRT or IGABT-based treatment. Rather, the value of this cohort is based on the rare opportunity it provides for direct radiological-pathological correlation between post-CCRT lymph node size and pathological nodal status in lymphadenectomy specimens. In this cohort, both the short-axis and long-axis diameters of the largest pelvic lymph node after CCRT were significantly larger in the ypN1 group than in the ypN0 group, and the short-axis diameter showed better overall diagnostic performance than the long-axis diameter.

After CCRT, enlarged or incompletely regressed pelvic lymph nodes are sometimes encountered, and it is often difficult to determine whether these findings represent treatment-related change or residual viable tumor. MRI and FDG-PET/CT have been widely used for post-treatment response assessment and surveillance in cervical cancer, but imaging findings obtained shortly after chemoradiotherapy may be affected by ongoing treatment-related changes, including edema, inflammation, necrosis, and fibrosis, which can lead to false-positive interpretation [11, 14]. Although previous studies have evaluated post-treatment MRI or PET/CT findings after CCRT and have reported the prognostic importance of pathological residual disease after chemoradiotherapy followed by surgery [18], direct radiological-pathological correlation of post-CCRT pelvic lymph nodes remains limited. In the present study, post-treatment nodal assessment was based on CT in 43 patients and MRI in 14 patients, which may have introduced measurement heterogeneity. Nevertheless, nodal size remains a simple and widely available imaging parameter. For lymph node assessment, the short-axis diameter is generally preferred because it is less influenced by nodal orientation and elongated morphology than the long-axis diameter [19]. Our results were consistent with this concept: the short-axis diameter showed a higher AUC than the long-axis diameter, suggesting that even after chemoradiotherapy—when nodal structure may be modified by treatment-induced fibrosis and partial volume loss—the short-axis diameter remains a more robust size-based indicator.

Regarding cutoff selection, the optimal threshold differed depending on the diagnostic index used. In the present analysis, a short-axis threshold of 5 mm yielded the highest Youden index, whereas 7 mm yielded the highest F1 score together with relatively favorable specificity (90.0%) and positive predictive value (71.4%). Notably, the candidate thresholds in our post-treatment cohort (5–7 mm) were lower than the conventional 10-mm short-axis threshold widely applied to untreated pelvic lymph nodes [12]. This may reflect the fact that the lymph nodes had already decreased in size after CCRT. Chemoradiotherapy reduces overall nodal size, so the size range that distinguishes residual disease from treatment-related change is correspondingly compressed. However, a decrease in lymph node size does not necessarily indicate the absence of viable tumor, and residual viable tumor may persist even in nodes that appear small on post-treatment imaging [10]. From a practical viewpoint, 7 mm may be easier to use when specificity is prioritized, whereas 5 mm may be preferable when sensitivity is prioritized.

Pre-treatment clinical nodal status (cN1) was the only baseline variable significantly associated with ypN1 disease in our cohort (*p* = 0.034). This finding aligns with the well-established prognostic significance of pre-treatment nodal involvement in cervical cancer, which was reinforced by the 2018 FIGO revision incorporating nodal disease into stage IIIC and which has motivated treatment-intensification strategies such as integrated or sequential nodal boost during definitive CCRT [17]. The result also suggests that the predictive value of post-CCRT lymph node size may be enhanced when interpreted in conjunction with pre-treatment clinical nodal status rather than as a stand-alone parameter. Clinically, patients with both cN1 disease at diagnosis and a residual short-axis diameter of ≥ 7 mm after CCRT may represent a higher-risk group in whom additional diagnostic evaluation—such as lymphadenectomy, FDG-PET/CT, or close imaging surveillance—may be reasonable, whereas patients with cN0 disease and a residual short-axis diameter < 5 mm may represent a lower-risk group.

The timing of post-treatment evaluation should also be considered when interpreting the present findings. In this cohort, surgery was planned after completion of CCRT, and post-treatment imaging was performed during the preoperative assessment period. The median interval from completion of CCRT to surgery was 26 days, and the median interval from post-treatment imaging to surgery was 16 days. Therefore, the present results reflect nodal status at a relatively early post-CCRT time point. At this stage, residual nodal enlargement may reflect not only viable tumor but also treatment-related edema, inflammation, or necrotic change [11, 12]. Previous reports have shown that post-treatment imaging assessment in cervical cancer is often performed at 3–6 months after chemoradiotherapy, and that treatment-related changes may persist for several months [20]. The antitumor effect of chemoradiotherapy may continue after treatment completion, and histological viability shortly after CCRT may not necessarily be equivalent to eventual clinical progression. Accordingly, the cutoff values identified in this study should be interpreted in the context of this early post-treatment evaluation window.

This study has several limitations. First, it was a retrospective analysis performed at a single institution, and the number of patients was relatively small. Second, the cohort was derived from a historical treatment strategy involving planned surgery after CCRT, which differs from current standard management using definitive chemoradiotherapy. Therefore, the proposed thresholds should not be regarded as clinically validated criteria for current practice, nor should they be directly applied to patients treated with current definitive CCRT. These thresholds should be considered hypothesis-generating estimates obtained from a selected historical cohort with direct radiological-pathological correlation. In addition, imaging protocols, scanner generations, and the timing of post-CCRT imaging varied across the 16-year study period, and inter-observer variability of nodal measurement was not formally assessed. Pre-treatment clinical nodal status was based on the treating physicians’ interpretation as documented in the medical records, which may have introduced inter-rater variability. External validation in independent cohorts will be required before any threshold can be recommended for routine clinical decision-making.

In conclusion, post-CCRT pelvic lymph node size, especially the short-axis diameter, was associated with pathological nodal status in this cohort. Although the present findings were obtained in a historical treatment setting, they suggest that residual nodal size on post-treatment imaging may be useful when estimating the likelihood of persistent nodal disease after CCRT, particularly when combined with pre-treatment clinical nodal status. Among the examined thresholds, 7 mm may be useful when specificity is prioritized, whereas 5 mm may be useful when a more balanced assessment is needed.

## Data Availability

The datasets analyzed during the current study are available from the corresponding author on reasonable request.
